# Mitochondria-targeted organic sonodynamic therapy agents: concept, benefits, and future directions

**DOI:** 10.3389/fchem.2023.1212193

**Published:** 2023-06-08

**Authors:** Eunbin Hwang, Minjae Yun, Hyo Sung Jung

**Affiliations:** ^1^ Department of Gerontology (AgeTech-Service Convergence Major), Graduate School of East-West Medical Science, Kyung Hee University, Yongin, Republic of Korea; ^2^ Department of Biomedical & Chemical Sciences, Hyupsung University, Hwasung, Republic of Korea

**Keywords:** sonodynamic therapy, mitochondria-targeting, organic-based agents, cancer therapy, reactive oxygen species

## Abstract

Sonodynamic therapy (SDT) is an emerging and potentially less invasive therapeutic approach for cancer that employs ultrasound (US)-sensitive agents combined with US irradiation to generate cytotoxic reactive oxygen species (ROS) in deep tumor regions. Among various cellular organelles, the mitochondria are particularly susceptible to ROS, making them an attractive target for SDT. Organic-based SDT agents with mitochondria-targeting affinity have gained considerable interest as potential alternatives to conventional SDT agents, offering significant advantages in the field of SDT. However, to date, a comprehensive review focusing on mitochondria-targeted SDT agents has not yet been published. In this review, we provide an overview of the general concept, importance, benefits, and limitations of mitochondria-targeted organic SDT agents in comparison to conventional SDT methods. Finally, we discuss the current challenges and future directions for the design and development of efficient SDT agents. By addressing these issues, we aim to stimulate further research and advancements in the field of mitochondria-targeted SDT, ultimately facilitating the translation of these agents into clinical applications.

## 1 Introduction

Sonodynamic therapy (SDT) represents a newly-emerging treatment modality in clinical settings that offers the possibility of less-invasive and selective eradication of deep-seated tumors (Son et al., 2020). SDT is based on ultrasound (US) irradiation of US-sensitive agents (also called SDT agents), that when in the presence of O_2_ or water, generates cytotoxic reactive oxygen species (ROS), thereby activating apoptosis of cancer cells. Simultaneously, the shear force activated by the US-mediated cavitation effect damages cancer cells through mechanical stress (Rengeng et al., 2017). While several studies have focused on finding or developing SDT agents, traditional SDT agents have shown limited therapeutic effects and safety concerns (Son et al., 2020; Zhang et al., 2021). Therefore, there is still a need for more advanced SDT agents and proven technologies for clinical application.

Organic-based agents offer satisfactory biocompatibility and are easy to prepare/modify, making them an attractive option to improve the therapeutic outcomes through enhanced or targeted cytotoxic ROS (Xing et al., 2021). However, major ROS typically have a very short half-life (singlet oxygen (^1^O_2_) and superoxide (•O_2_): 10^−6^ s; hydroxyl radical (•OH): 10^−9^ s) (Rubio and Cerón, 2021), limiting their action to sites of production (<20 nm), which are much smaller than the size of cancer cells (Kim et al., 2014) This poses a significant challenge that needs to be overcome to fully harness the benefits of SDT. Therefore, delivering organic SDT agents to cancer cells, particularly to critical subcellular organelles vulnerable to ROS, could be a promising strategy to improve SDT therapeutic outcomes. Considering this, substantial efforts have been devoted to studying of new organic SDT agents, especially those capable of delivering ROS to specific affected biological sites, such as mitochondria, in a precise manner (Fulda et al., 2010).

Mitochondria, as preferential organelle targets for improved therapeutic outcomes, not only play a key role in tumor apoptosis or survival but are also highly susceptible to excessive ROS generated by SDT (Lockman et al., 2010; Bacellar et al., 2019; Chen et al., 2019). Mitochondrial-targeted US-sensitive organic agents have recently gained attention as a potential complements to traditional agents, offering important advantages such as enhanced antitumor efficacy (Zhang et al., 2020; Li et al., 2022), activation of antitumor immune responses (Ren et al., 2021; Shi et al., 2023), and overcoming multiple SDT challenges (e.g., metastasis) (Han et al., 2021; Ji et al., 2021). These agents can also serve as multimodal approaches, combining SDT with other imaging-guided modalities such as photoacoustic (PA) imaging, magnetic resonance (MR) imaging, fluorescence (FL) imaging, etc. (Zhang et al., 2019; Ren et al., 2021). Targeting ligands in the mitochondria of cancer cells involves triphenylphosphonium (TPP) (Song et al., 2019), which has been applied in SDT agents utilizing hematoporphyrin monomethyl ether (HMME) (Chen et al., 2017), chlorin e6 (Ce6) (Cao et al., 2023), among others. as the sonodynamic payload. Furthermore, some US-sensitive agents, such as IR780 (Zhang et al., 2019) and protoporphyrin IX (PpIX) (Xie et al., 2017), as well as adipocyte-derived organelle lipid droplets (LDs) (Shi et al., 2023), with inherent mitochondria-targeting affinity, are being investigated for this purpose.

**FIGURE 1:**
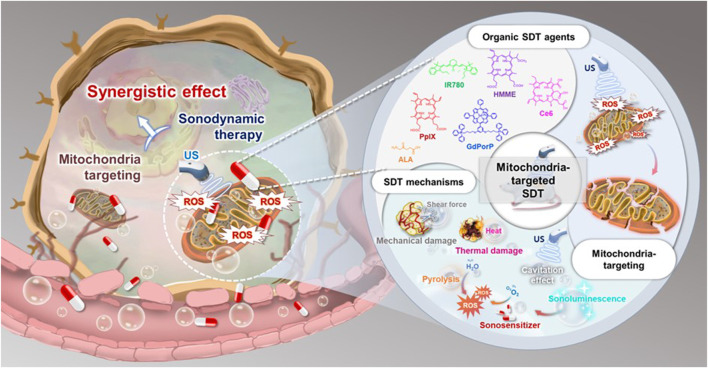
Overview of mitochondria-targeted organic SDT agents.

Ideally, mitochondria-targeted organic SDT agents should meet the following significant requirements (Figure 1): 1) achieving excellent anticancer efficacy by selecting high-efficiency organic SDT agents; 2) appropriately modifying the agents using suitable methods for US-mediated ROS generation targeting both tumor cells and specific mitochondria, through modifications in the composition of the agents, introducing mitochondria-targeting moieties to the agents and creating activatable agents for mitochondria specific environments; 3) minimizing the dark toxicity of agents; 4) rapid disintegration after SDT application to avoid adverse effects; and 5) easy preparation and modification at a low cost.

Despite several reports on newly-emerging agents with mitochondrial affinity in recent years, no comprehensive review papers regarding these agents have been published thus far. Therefore, this review focuses on the recent advances in mitochondrial SDT agents, covering their general principle, importance, benefits, and challenges compared to the traditional SDT methods. We herein summarize the agent types, experimental conditions, *in vitro* anticancer efficacies, and their therapeutic strategies, as shown in Table 1.

**TABLE 1:** Mitochondria-targeted organic SDT agents for cancer therapy.

SDT agent	Probe construction	US irradiation	Cell viability (off-target vs. mitochondria-targeted SDT)	Therapeutic strategy	Reference
IR780	PFP/IR780 core-shell nanodroplets (IR780-NDs)	3 min	<40% for IR780-NDs vs. >90% for NDs (w/o IR780) cell viability of 4T1 cells at 121 μg/mL	Combination of mitochondria-targeted SDT, deep penetration, and FL/PA imaging visualization	Zhang et al. (2019)
		650 kHz			
		2.4 W/cm^2^			
IR780	R162/IR780 TPP-based lipid bilayer (MLipRIR NPs)	1 min	16.4% for MLipRIR NPs vs. 27.6% for LipRIR NPs (w/o TPP) cell viability of 4T1 cells at 1 μg/mL (R162 conc.)	Combination of mitochondrial SDT and immunotherapy (w/R162)	Ren et al. (2021)
		1.0 MHz			
		1.5 W/cm^2^			
		50% duty cycle			
IR780	HSA-NO/FDC nano-formulation (PIH-NO)	1 min	30% for PIH-NO vs. 50% for PIH (w/o HSA-NO) and 70% for IH (w/o HSA-NO and FDC) cell viability of 4T1 cells at 4 μg/mL (IR780 conc.)	Combination of mitochondria-targeted SDT and gas therapy (w/HSA-NO and FDC)	Ji et al. (2021)
		1.0 MHz			
		1.5 W/cm^2^ 50% duty cycle			
IR780	IR780/CPI-613/adipocyte-derived organelle LDs (CPI-613/IR780@LDs)	1 min	<40% for CPI-613/IR780@LDs cell viability of both normoxic and hypoxic Panc02 cells at 20 μmol/L	Combination of mitochondria-targeted SDT and metabolic modulation therapy (w/CPI-613)	Shi et al. (2023)
		1 MHz			
		0.5 W/cm^2^			
		50% duty cycle			
ALA	ALA/2-DG/MB	5 min	33.2% for ALA/2-DG/MB vs. 49.2% for ALA/2-DG cell viability of HepG2 cells at 2 mmol/L (2-DG)	Combination of mitochondria-targeted SDT, metabolic modulation therapy (w/2-DG), and US-stimulated microbubble therapy (w/MB)	Xie et al. (2017)
		3 MHz			
		2 W/cm^2^			
		60% duty cycle			
HMME	HMME/TPP-Chol liposome (HMME-Lipo-TPP)	3 min	∼32% for HMME-Lipo-TPP vs. ∼44% for HMME-Lipo (w/o TPP) cell viability of MCF-7 cells at 20 μg/mL	US-assisted increased cancer cells mitochondria delivery	Chen et al. (2017b)
		1 MHz			
		0.5 W/cm^2^			
Ce6	TPP-Ce6/PL/EV (EV (TPP-Ce6/PL)	1 min	∼6.5% for EV (TPP-Ce6/PL) vs. ∼30% for EV (TPP-Ce6) and ∼70% for TPP-Ce6 cell viability of MCF-7 cells at 10 μM (TPP-Ce6 (or Ce6) conc.)	Combination of mitochondria-targeted SDT and chemotherapy (w/PL)	Cao et al. (2023)
		1 MHz			
		0.3 W/cm^2^			
IR780/HMME	IR780/HMME/GOx/PLGA core-shell nano-formulation (IHG@P)	30 s	8.48% for IHG@P vs. ∼68% for starvation therapy alone and ∼58% for SDT therapy alone cell viability of 4T1 cells at 60 μg/mL (PLGA conc.)	Combination of mitochondria-targeted dual SDT agent therapy, starvation therapy (w/GOx), and FL/PA imaging visualization	Zhang et al. (2020)
		2 W/cm^2^			
IR780/Ce6	PEG-IR780/Ce6 nano-micelle (PEG-IR780@Ce6)	3 min	35% for PEG-IR780@Ce6 vs. 85% for free Ce6 and 43% for PEG-IR780 cell viability of MDA-MB-231 cells at 40 μg/mL	Mitochondria-targeted dual SDT agent therapy	Han et al. (2021)
		1 MHz			
		0.6 W/cm^2^			
		50% duty rate)			
Gd(III)- porphyrinate	TPP-Gd(III) porphyrinate/MAL-PEG-*b*-PDPA pH-responsive nanomicelle (RPGdP)	1 min	25% for RPGdP vs. 65% for RPGd (w/o TPP) cell viability of H22 cancer cells at 80 μg/mL	Combination of mitochondria-targeted SDIT and MR imaging visualization	Li et al. (2022)
		1 MHz			
		1.5 W/cm^2^			

[Abbreviation] PFP: perfluoropentane, NDs: nanodroplets, w/o: without, PA: photoacoustic, FL: fluorescence, MR: magnetic resonance, R162: glutamate dehydrogenase 1 inhibitor, TPP: triphenylphosphonium, conc.: concentration, HSA-NO: human serum albumin-based NO donor, FDC: perfluorodecalin, CPI-613: FDA-approved TCA cycle inhibitor, LDs: lipid droplets, w/: with, ALA: 5-aminolevulinic acid, 2-DG: glycolysis inhibitor 2-deoxyglucose, MB: microbubble, HMME: hematoporphyrin monomethyl ether, Chol: cholesterol, Ce6: Chlorin e6, PL: prooxidant piperlongumine, EV: extracellular vesicle, GOx: glucose oxidase, PLGA: polylactic-co-glycolic acid, SDIT: sonodynamic immunotherapy SDT: sonodynamic therapy.

## 2 Working principle of sonodynamic therapy

SDT agents can be activated by several mechanisms, including light-induced sonoluminescence (SL), increased acoustic cavitation, and pyrolytic reactions. During SDT, ultrasonic waves create cavitation bubbles that absorb the energy of the sound waves. As the energy is released, the bubbles collapse, generating a high-pressure-high-temperature environment of over 81 MPa and 10,000 K in the affected area.

First, this phenomenon produces SL, which can excite the electron orbitals of the SDT agent via sound energy transfer; SL can generate electron holes, which can subsequently pair with the generated ROS, such as •O_2_, •OH, or ^1^O_2_ (Saksena and Nyborg, 1970).

Second, increased acoustic cavitation directly excites the agents to generate two types of ROS by different reactions (Costley et al., 2015; Canavese et al., 2018): 1) the energy released from excited SDT agents to the singlet ground state can be transferred to ambient oxygen to generate ^1^O_2_, which is considered a major mediator of SDT; 2) excited-state SDT agents can react directly with other adjacent substrates or oxygens to convert hydrogen atoms into free radicals.

Third, the collapse of cavitation bubbles also provides enough energy to pyrolyze water and subsequently generate hydroxyl radicals (•OH) (Riesz et al., 1992). Furthermore, the locally elevated temperature and the cavitation energy can decompose the SDT agent and generate free radicals, such as •O_2_ and •OH (Qian et al., 2016).

Ultimately, the produced ROS causes increased oxidative stress, resulting in irreversible cellular damage. Moreover, the shear forces and heat generated by the collapse of cavitation bubbles in the focal region result in severe mechanical and heat-shock damage to the cell membrane and cytoskeleton, culminating in cancer cell apoptosis.

## 3 General principle of mitochondria targeting

Mitochondria, commonly referred to as the cell’s powerhouse, are double-membrane cell organelles with their own double-stranded circular mitochondrial DNA (mtDNA) (Murphy et al., 2018). The mitochondrial transmembrane potential (MTP) value is around −140 mV in the normal cell inner membrane, while it is significantly increased in tumor cells (∼-220 mV) (Huang et al., 2021). Therefore, SDT agents with strong delocalized positive potential and lipophilicity can effectively cross the cancer mitochondrial lipid bilayer membrane and accumulate within the mitochondria by hundreds of folds due to the high negative inner membrane potential. Mitochondrial disruption regulates the endogenous pathway to apoptosis, promoting caspase 3 expression and the production of cytochrome c, the main effectors of cell apoptosis (Bhatti et al., 2017).

Precise targeting of cancer cell mitochondria using SDT agents has already been accomplished in several studies (Chen et al., 2017; Xie et al., 2017; Zhang et al., 2019; Zhang et al., 2020; Han et al., 2021; Li et al., 2022; Cao et al., 2023). TPP, with a strong positive potential and lipophilicity, is the most frequently used agent for this purpose (Song et al., 2019). Additionally, a few US-sensitive agents such as IR780 (Zhang et al., 2019) and PpIX (Xie et al., 2017), and adipocyte-derived organelle LDs (Shi et al., 2023), with their inherent targeting ability, are being explored. Moreover, a variety of agents can be delivered to the mitochondria for SDT using various techniques (Li and Huang, 2020). For example, small molecule ligands such as glycyrrhetinic acid and guanidine can be used, as well as mitochondrial penetrating peptides, Szeto–Schiller peptides, and mitochondrial targeting sequences. Additionally, vehicle-type mitochondrial targeted systems such as MITO-Porter vehicles can be utilized for this purpose.

## 4 Mitochondria-targeted organic SDT agents

### 4.1 IR780-based SDT agents

IR780 is a lipophilic heptamethine cyanine agent with lipophilic properties that has been clinically employed in FL/PA imaging-guided procedures (Li et al., 2018). IR780 exhibits an inherent ability to target tumor cells and mitochondria, allowing for deep tumor penetration without the need for additional mitochondria-targeting moieties due to its lipophilic cationic property (Wang et al., 2014). Additionally, IR780 demonstrates significant SDT properties (Chen et al., 2017); however, it is characterized by limited water solubility and poor photostability. Despite these limitations, the combination of IR780 with FL/PA imaging approaches holds promise for imaging-guided SDT in deep tumors (Cheng et al., 2015).

**FIGURE 2:**
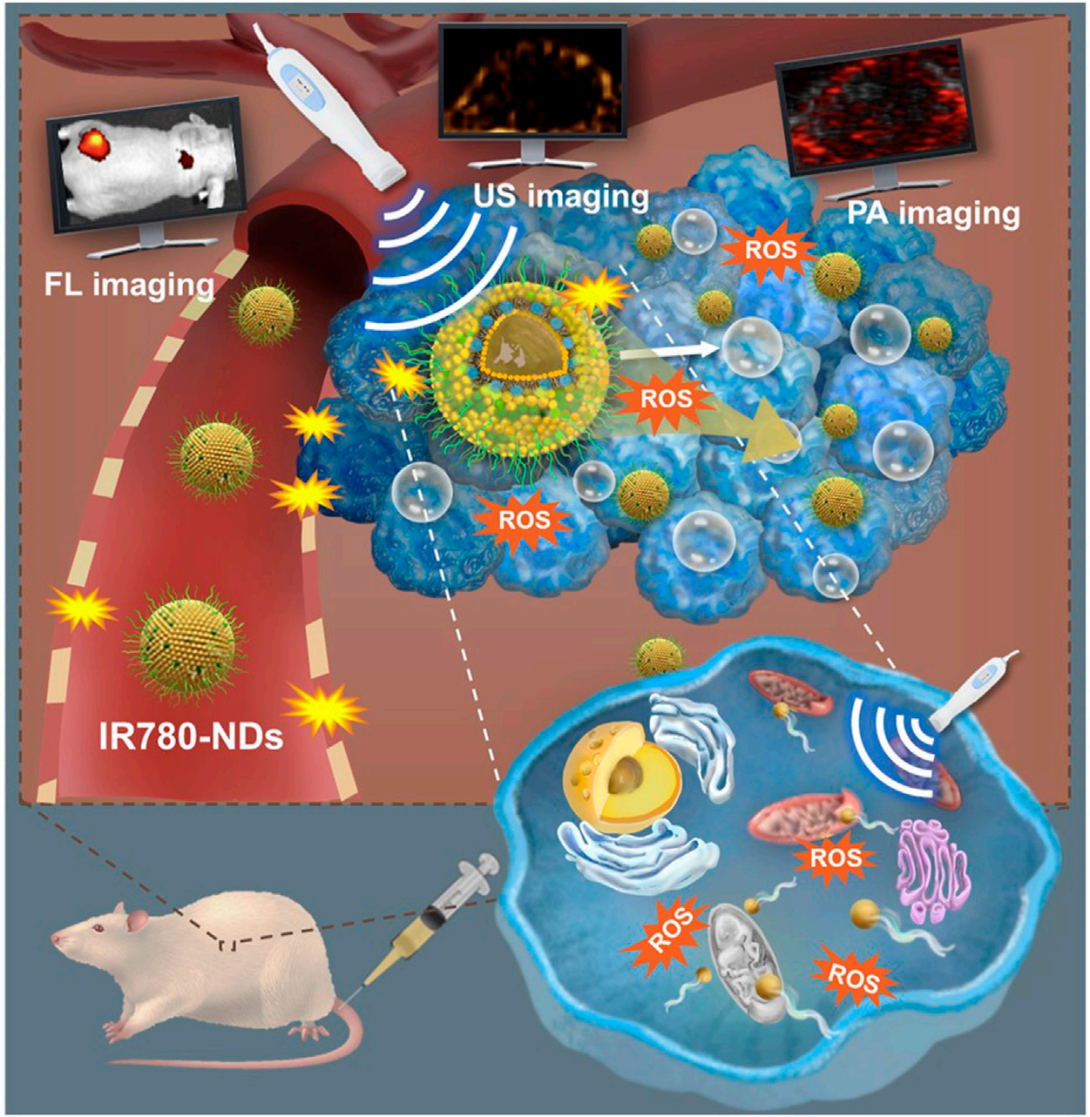
Schematic illustration of US-mediated multifunctional IR780-NDs SDT system for deep penetration, mitochondrial targeting, and simultaneous FL/PA/US imaging. Reprinted with permission from ref (Zhang et al., 2019). Copyright (2019) American Chemical Society.

In 2019, Zhang et al. (2019) developed IR780-loaded nanodroplets (IR780-NDs) with a well-designed core-shell structure (core: perfluoropentane (PFP); shell: IR780), enabling a US-mediated multifunctional IR780-NDs SDT system with deep penetration, mitochondrial targeting, and simultaneous FL/PA/US imaging abilities (Figure 2). Under US irradiation (3 min, 650 kHz, 2.4 W/cm^2^), IR780-NDs penetrated from the tumor surface to its core through the acoustic droplet vaporization (ADV) effect of PFP (Mura et al., 2013; Ho et al., 2017), demonstrating excellent depth of penetration. In comparison to NDs (without IR780), IR780-loaded NDs selectively accumulated in and were activated by mitochondria (0.87 vs. −0.15 Pearson coefficient value, respectively). This targeted accumulation can induce oxidative stress through US-mediated ROS production and resulted in apoptosis of 4T1 cells (<40% vs. >90% cell viability, respectively, at 121 μg/mL). Moreover, the efficacy of IR-780 NDs was evaluated in 4T1 tumor mouse models, demonstrating enhanced SDT, including FL/US/PA imaging abilities, likely attributed to the mitochondria-targeted SDT effect in cancer cells and amplified by the unique ADV effect of PFP.

**FIGURE 3:**
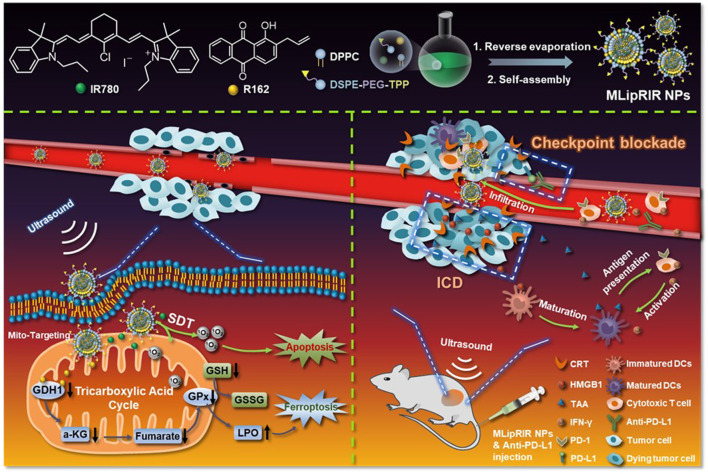
Schematic illustration of mitochondrial-targeting liposomal nanoparticles (MLipRIR NPs) for both FL/PA multimodal imaging and SDT reinforced by ICD. Adapted with permission from ref. (Ren et al., 2021). 2021, Ivyspring International Publisher.

Immunogenic cell death (ICD) is a form of cell death that stimulates the release of tumor-specific antigens and danger-associated molecular patterns (DAMPs), triggering a systemic antigen-mediated antitumor immune response (Krysko et al., 2012). Recent studies have suggested that ICD has the potential to enhance the efficacy of SDT (Yue et al., 2019; Li X. et al., 2022). In 2021, Ren et al. (2021) employed mitochondrial-targeting liposomal nanoparticles (MLipRIR NPs) to combine FL/PA multimodal imaging and SDT, reinforced by ICD (Figure 3). The NPs were developed by encapsulating R162 (glutamate dehydrogenase 1 inhibitor) and IR780 within a mitochondria-targetable lipid bilayer, efficiently entering the 4T1 cell and specifically targeting the mitochondria through a TPP moiety modification on the NPs surface. Under US irradiation, IR780 released from MLipRIR NPs generated high levels of ROS, impeding mitochondrial respiration and leading to local glutathione (GSH) consumption. In addition, the co-released R162 inhibited glutamate dehydrogenase 1 (GDH1) by interfering with the anaplerotic use of glutamine in the mitochondrial TCA cycle (Jin et al., 2015). These effects induced ferroptosis of cancer cells through lipid peroxide accumulation, effectively activating ICD. MLipRIR NPs also demonstrated superior US-mediated cytotoxicity in 4T1 cells (16.4% cell viability; R162 concentration = 1 μg/mL) relative to LipRIR NPs without TPP moiety modification (27.6% cell viability; R162 concentration = 1 μg/mL) under US irradiation (1 min, 1.0 MHz, 1.5 W/cm^2^, 50% duty cycle). By leveraging the bimodal imaging capabilities of MLipRIR NPs, *in vivo* FL/PA multimodal imaging was successfully conducted in the 4T1 mouse models. Furthermore, studies in 4T1 mouse models showed that MLipRIR NPs efficiently reduced tumor size and mitigated severe side effects of cancer treatment on other tissues.

**FIGURE 4:**
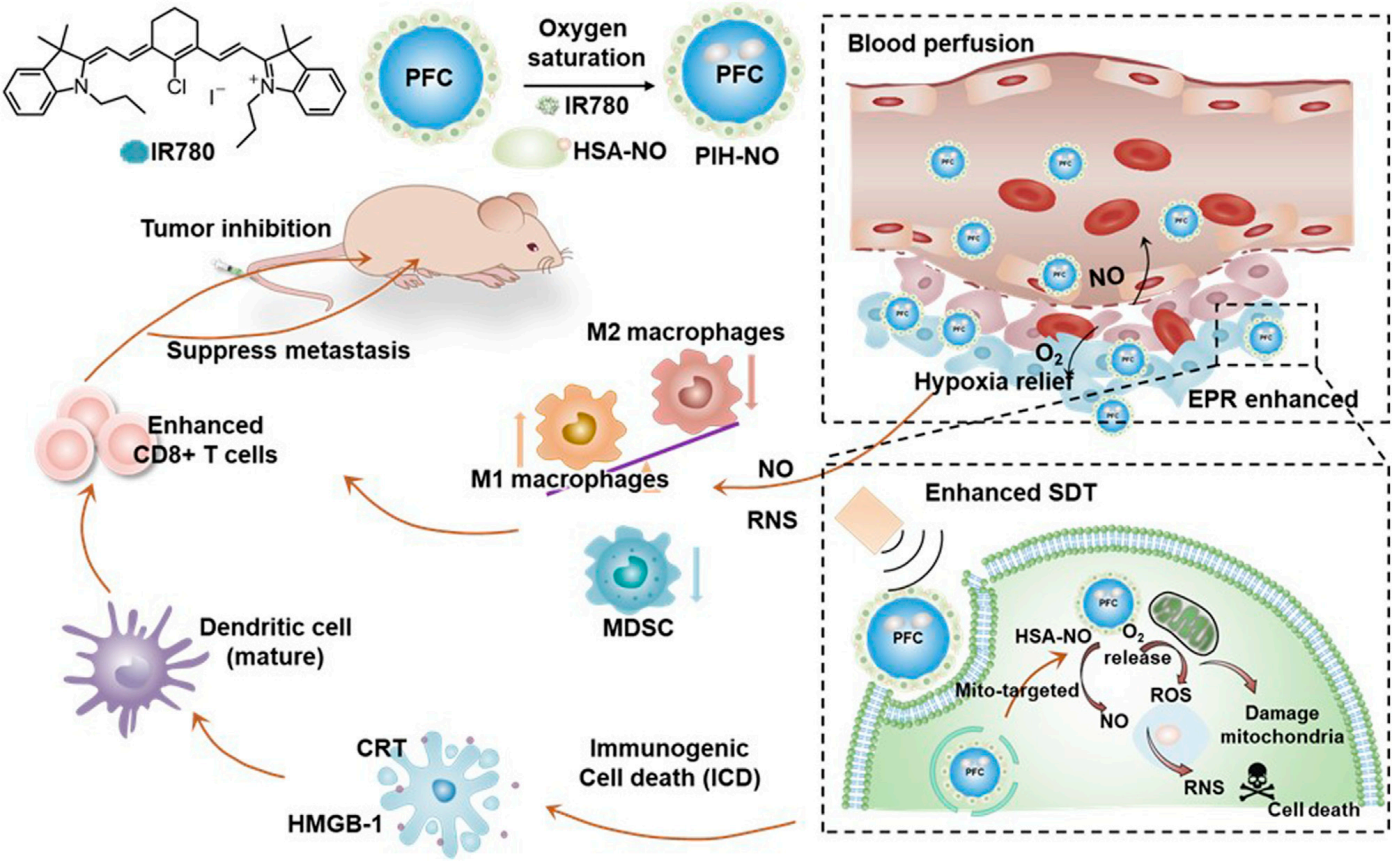
Schematic illustration of mitochondria-targeted nano-formulations (PIH-NO) for effective cancer sonodynamic immunotherapy. Adapted with permission from ref. (Ji et al., 2021). 2021, Ivyspring International Publisher.

Ji et al. (2021) developed mitochondria-targeted formulations (PIH-NO) by encapsulating IR780 in a human serum albumin-based NO donor (HSA-NO) and an oxygen-carrier perfluorodecalin (FDC), a type of perfluorocarbon (PFC) (Figure 4). PIH-NO accumulated in the 4T1 cell mitochondria (PC = 0.76), leading to mitochondrial dysfunction, relief of hypoxia, and amplified ICD through ROS production, enhanced by the explosive release of NO and O_2_ upon US irradiation. Notably, significant cytotoxicity was observed in the PIH-NO (30% cell viability; equivalent IR780 concentration = 4 μg/mL) compared to PIH (without NO donor) (50% cell viability; equivalent IR780 concentration = 4 μg/mL) and IH (without NO donor and oxygen-carrier) (70% cell viability; equivalent IR780 concentration = 4 μg/mL) under US irradiation conditions (1 min, 1.0 MHz, 1.5 W/cm^2^, 50% duty cycle). Furthermore, observations in 4T1 mouse models indicated that PIH-NO effectively reduced tumor size and significantly decreased the number of pulmonary metastatic nodules. Hence, this study demonstrated the potential of gas transportation to amplify mitochondrial SDT, enhancing its therapeutic efficacy, inducing anticancer immune responses and preventing metastasis.

**FIGURE 5:**
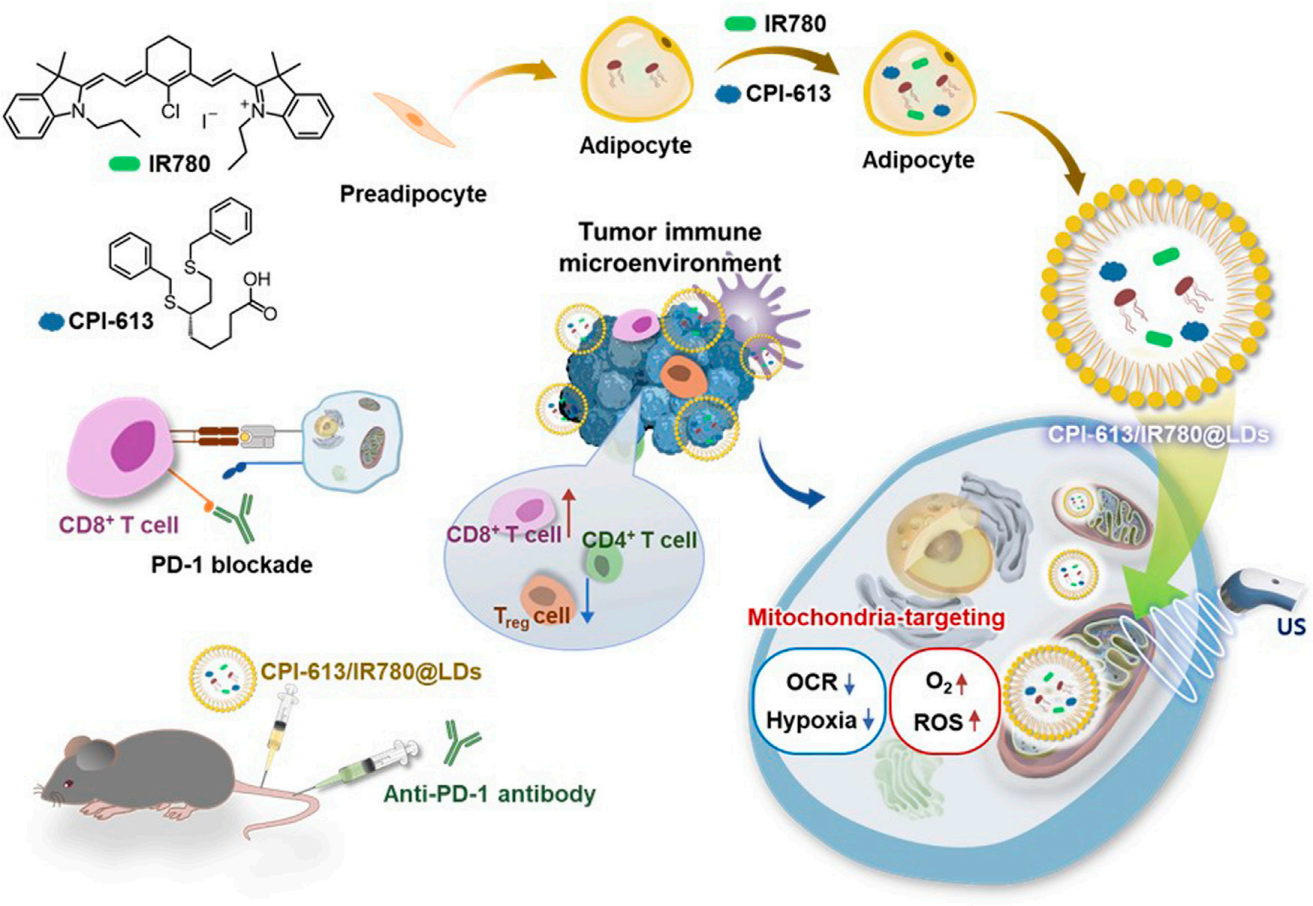
Schematic illustration of a novel approach for mitochondrial SDT using adipocyte-derived organelle LDs as carrier agents with an inherent affinity for mitochondria.

Shi et al. (2023) developed a novel approach for mitochondrial SDT using adipocyte-derived organelle LDs as carrier agents with an inherent affinity for mitochondria (Figure 5). The developed LDs (CPI-613/IR780@LDs), contained IR780 and CPI-613, a FDA-approved TCA cycle inhibitor. By synergistically inhibiting the mitochondrial TCA cycle and enhancing ROS production at the tumor site, the combination of mitochondrial SDT and CPI-613 enabled efficient mitochondrial SDT in Panc02 tumors. Additionally, combining mitochondrial SDT and anti-PD-1-mediated immunotherapy reinforced the immune response in Panc02 tumors. CPI-613/IR780@LDs demonstrated significantly increased cell cytotoxicity, overcoming the limited efficacy of SDT induced by the hypoxia in the tumor microenvironment (TME) after US irradiation (1 min, 1 MHz, 0.5 W/cm^2^, 50% duty cycle) in both normoxic and hypoxic Panc02 cells (<40% cell viability of both normoxic and hypoxic Panc02 cells; CPI-613 concentration: 154.24 μmol/L, and IR780 concentration: 20 μmol/L). This approach successfully attenuated the progression of primary and metastatic tumors in Panc02-bearing mouse models.

### 4.2 5-Aminolevulinic acid-based SDT agent

The 5-aminolevulinic acid (ALA) is a powerful SDT agent that is converted to PpIX and delivers it to the heme synthetic root of cancer mitochondria. Previous studies have explored the use of ALA-based SDT for various cancers (Li et al., 2015; Wu et al., 2019).

Cancer cells rely on two main pathways for the energy production: mitochondrial aerobic oxidation and cytoplasmic anaerobic glycolysis (Fu et al., 2019a; Fu et al., 2019b). Mitochondria-targeted SDT inhibits the mitochondrial aerobic oxidation process by generating an excessive amount of ROS, thus impacting tumor cell growth. However, this approach alone may have limitations because tumor cells can still obtain energy through anaerobic glycolysis (Wang et al., 2017). To address this, Xie et al. (2017) demonstrated the synergistic effect of combining ALA, a precursor of PpIX, the glycolysis inhibitor 2-deoxyglucose (2-DG), and microbubbles (MBs) . Upon cell internalization, PpIX induced by ALA selectively accumulated in the mitochondria of the HepG2 cells. Furthermore, US irradiation (5 min, 3 MHz, 2 W/cm^2^, 60% duty rate) resulted in a potentiated therapeutic outcome with the combination system compared to both the ALA combined with 2-DG and MB (33.2%; 2-DG concentration = 2 mmol/L) and the ALA combined with 2-DG alone (49.2%: 2-DG concentration = 2 mmol/L). This study demonstrated that SDT enhanced with the glycolytic inhibitor 2-DG induces cell death in HepG2 cells, and MB enhances the outcome of SDT. However, further research is needed to investigate SDT application *in vivo*.

### 4.3 Hematoporphyrin monomethyl ether-based SDT agent

HMME, a derivative of PpIX, is commonly used as a photodynamic agent. However, it can also be activated by US irradiation to effectively generate ROS for SDT (Tian et al., 2009). Due to its hydrophobic nature, HMME is typically loaded into liposomes.

**FIGURE 6:**
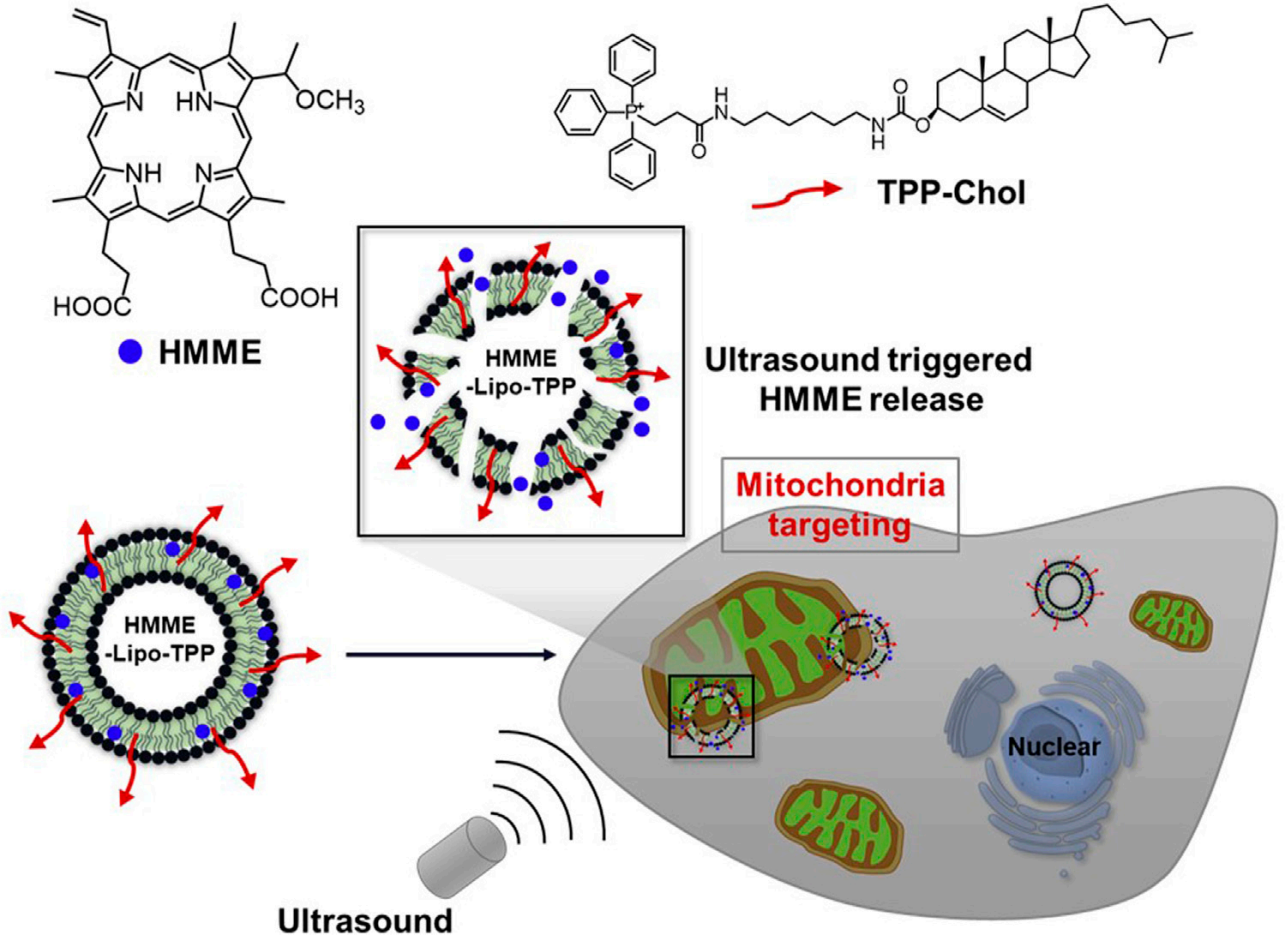
Schematic illustration of a mitochondria-targeting liposomal formulation of HMME modified with TPP (HMME-Lipo-TPP) for effective SDT. Reprinted with permission from (Chen et al., 2017), Copyright 2017 Elsevier.

Chen M. et al. (2017) developed a mitochondria-targeting liposomal formulation of HMME modified with TPP (HMME-Lipo-TPP) and investigated its efficacy for SDT (Figure 6). The formulation specifically targets mitochondria in MCF-7 cells and rapidly releases HMME upon US irradiation (3 min, 1 MHz, 0.5 W/cm^2^), leading to the production of ROS and induction of cell apoptosis. The cytotoxicity of HMME-Lipo-TPP in MCF-7 cells was superior to that of non-targeted controls (HMME-Lipo) under similar experimental conditions (∼32% vs. ∼44% cell viability, respectively, at 20 μg/mL). Although the *in vitro* characteristic of HMME-Lipo-TPP outperformed those of HMME-Lipo, further studies *in vivo* are required to validate its efficacy.

### 4.4 Chlorin e6-based SDT agent

Ce6 is a second-generation SDT agent that has been widely used to overcome the limitations of conventional agents, such as limited tumor targeting ability and slow clearance from normal tissues (Klyta et al., 2011). However, Ce6 still has drawbacks, including inefficient intracellular accumulation and low tumor specificity, limiting its SDT efficacy.

**FIGURE 7:**
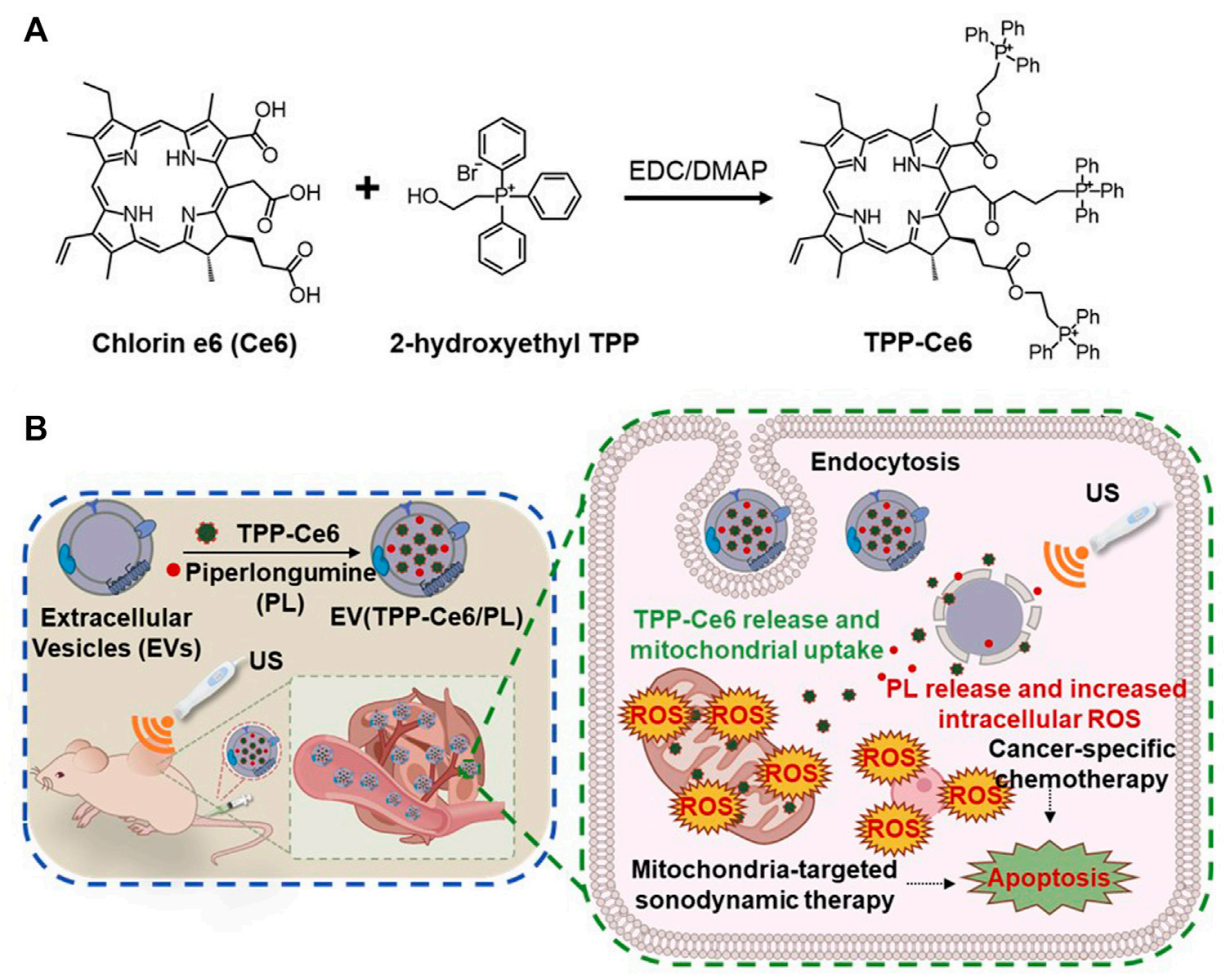
**(A)** Synthetic pathway of TPP-Ce6. **(B)** Schematic illustration of the mitochondria-targeted Ce6/PL-loaded extracellular vesicle (EV) (EV (TPP-Ce6/PL)) for biocompatible and efficient mitochondria-targeting chemo-SDT. Reprinted with permission from (Cao et al., 2023), Copyright 2023 Elsevier.

Cao et al. (2023) used a mitochondria-targeted approach using Ce6-loaded extracellular vesicles (EVs) (EV (TPP-Ce6)) to create a biocompatible and efficient mitochondria-targeting SDT (Figure 7). EVs have demonstrated clinical applicability as effective carriers for delivering SDT agents to tumor cells (Kalluri et al., 2020; Moller, 2020). In this study, Ce6 was conjugated with a TPP ligand and encapsulated within EVs. This formulation significantly increased the mitochondrial uptake of Ce6 (0.58 for EV (TPP-Ce6) vs. 0.31 for Ce6 Mander’s overlap coefficient). Under US irradiation (1 min, 1 MHz, 0.3 W/cm^2^), EV (TPP-Ce6) exhibited higher sono-cytotoxicity in MCF-7 cells than Ce6 alone (∼30% for EV (TPP-Ce6) vs. ∼70% for TPP-Ce6 cell viability; TPP-Ce6 (or Ce6) concentration = 10 μM, EVs concentration = 4.96 × 10^10^ EVs/mL). Furthermore, the co-encapsulation of prooxidant piperlongumine (PL) into EV (TPP-Ce6) demonstrated the most effective tumor inhibition through the synergistic effect of chemo-SDT under similar experimental conditions (∼6.5% cell viability; TPP-Ce6 (or Ce6) concentration = 10 μM, PL concentration = 3 μg/mL, EVs concentration = 4.96 × 10^10^ EVs/mL). Animal studies in MCF-7 cell mouse models demonstrated that this system significantly reduced tumor growth and, importantly, minimized side effects on other tissues.

### 4.5 IR780/hematoporphyrin monomethyl ether-based SDT agent

**FIGURE 8:**
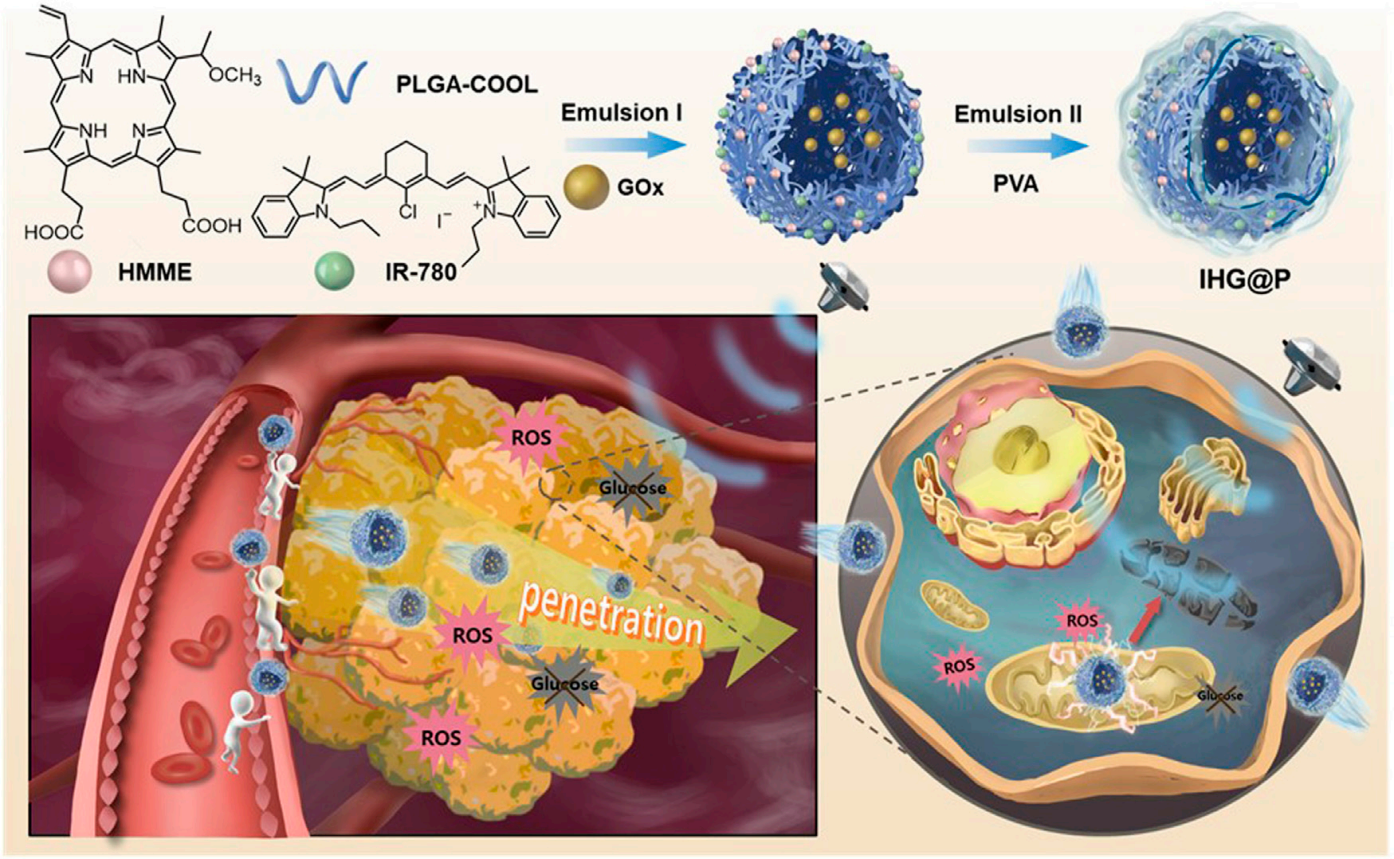
Schematic illustration of the core/shell structured PLGA nano-formulations (IHG@P), consisting of two sono-sensitizing agents (IR780 and HMME) and GOx, combining mitochondria-targeted dual SDT agent therapy, starvation therapy, and FL/PA imaging visualization. Reprinted with permission from Wiley (Zhang et al., 2020).

Zhang et al. (2020) reported a synergistic approach combining FL/PA bimodal imaging, mitochondria-targeted SDT, and starvation therapy using a combination of IR780 and HMME for FL/PA imaging and SDT and glucose oxidase (GOx) for starvation therapy (Figure 8). The system employed core/shell structured polylactic-co-glycolic acid (PLGA) nano-formulations (IHG@P), which consisted of GOx in the core and IR780 and HMME in the shell. This nano-formulation efficiently penetrated into 4T1 tumor cells, targeting the mitochondria, due to the inherent affinity of IR780 (PC = 0.806). As an adjuvant therapy, the GOx-mediated starvation therapy effectively suppressed cytosolic anaerobic glycolysis, limiting the energy supply of cancer cells and inducing tumor apoptosis. Under US irradiation (30 s, 2 W/cm^2^), the IHG@P group (8.48% Cell viability; PLGA concentration = 60 μg/mL) demonstrated the most significant 4TI cancer cell apoptosis compared to the groups receiving starvation therapy alone (∼68% cell viability; PLGA concentration = 60 μg/mL) or SDT therapy alone (∼58% cell viability; PLGA concentration = 60 μg/mL) groups. Additionally, IHG@P was used for *in vivo* FL/PA multifunctional imaging of 4T1 cell mouse models, showing similar synergistic effects of SDT and starvation therapy.

### 4.6 IR780/chlorin e6-based SDT agent

**FIGURE 9:**
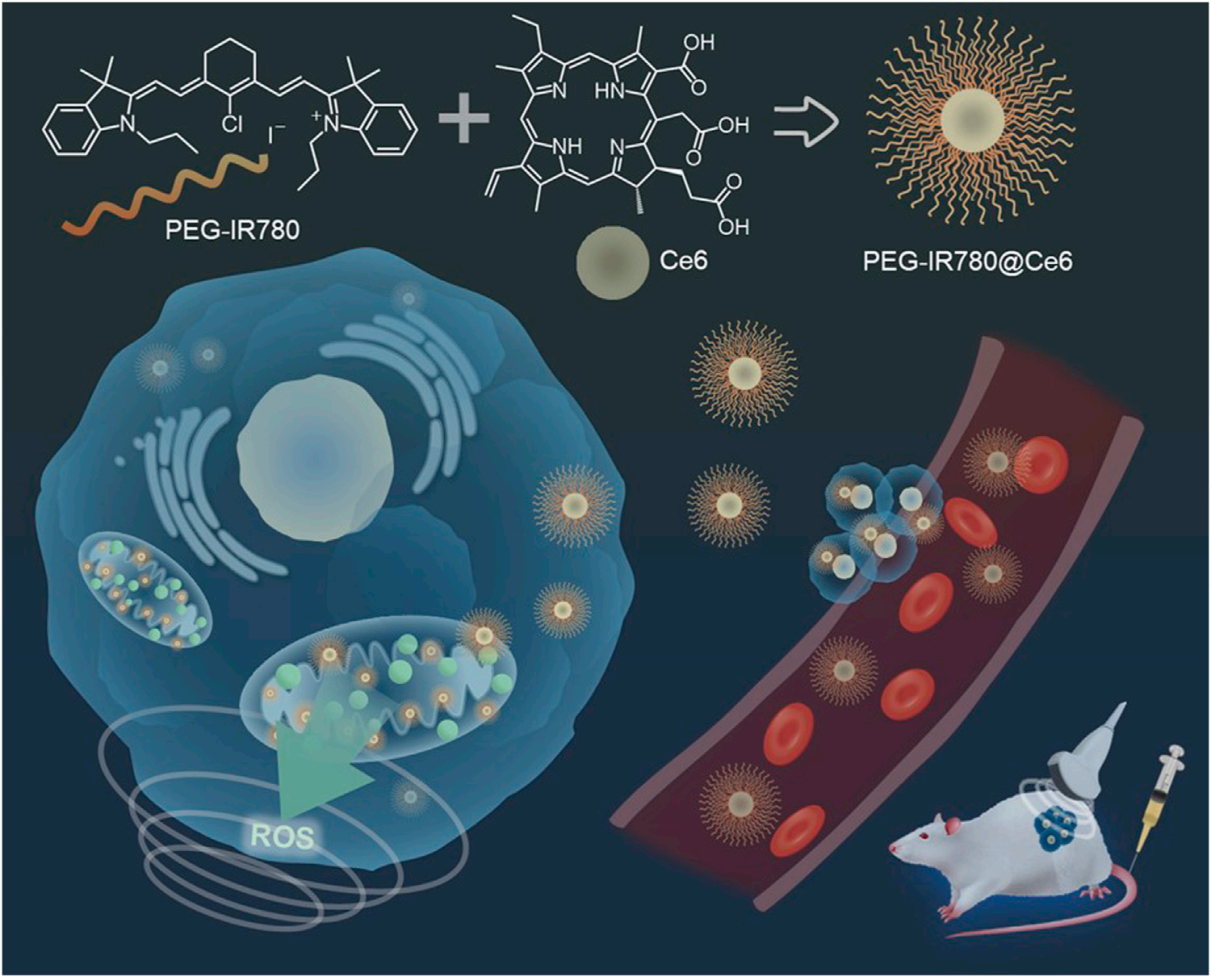
Schematic illustration of the new type of SDT complex nano-micelle (PEG-IR780@Ce6) for the treatment of triple-negative breast cancer (TNBC). Reprinted with permission from (Han et al., 2021), Copyright 2021 Elsevier.

Triple-negative breast cancer (TNBC) is a highly aggressive breast cancer subtype with limited treatment options and a high metastasis rate, posing considerable challenges for effective therapy (O’Reilly et al., 2015). To address this issue, Han et al. (2021) developed a novel SDT complex nano-micelle (PEG-IR780@Ce6) for the efficient treatment of TNBC. This nano-micelle combines two sono-sensitizing agents, PEGylated IR780 (PEG-IR780) and Ce6 (Figure 9) and exhibited enhanced uptake by MDA-MB-231 TNBC cells compared to PEG-IR780 or free Ce6 alone. The nano-micelle was efficiently delivered and accumulated in the mitochondria of MDA-MB-231 cells, resulting in ROS generation at the tumor site upon US irradiation. *In vitro* SDT experiments demonstrated the superior anticancer effects of PEG-IR780@Ce6 compared to PEG-IR780 or free Ce6 alone, highlighting its potential for sonodynamic TNBC therapy (3 min, 1 MHz, 0.6 W/cm^2^, 50% duty rate) (35% for PEG-IR780@Ce6 vs. 85% for free Ce6 and 43% for PEG-IR780 cell viability, at 40 μg/mL). Furthermore, *in vivo* SDT results demonstrated that PEG-IR780@Ce6 effectively eradicated TNBC tumors, along with significant suppression of the invasion and migration, while exhibiting minimal side effects.

### 4.7 Gadolinium (III) porphyrinate-based SDT agent

To achieve effective sonodynamic immunotherapy (SDIT), it is crucial to enhance the level of ICD induction of current SDT agent-based inducers (Zhang et al., 2018). Therefore, the development of a new SDT agent with the potential to promote high ROS production and with mitochondrial targeting ability is timely and necessary.

**FIGURE 10:**
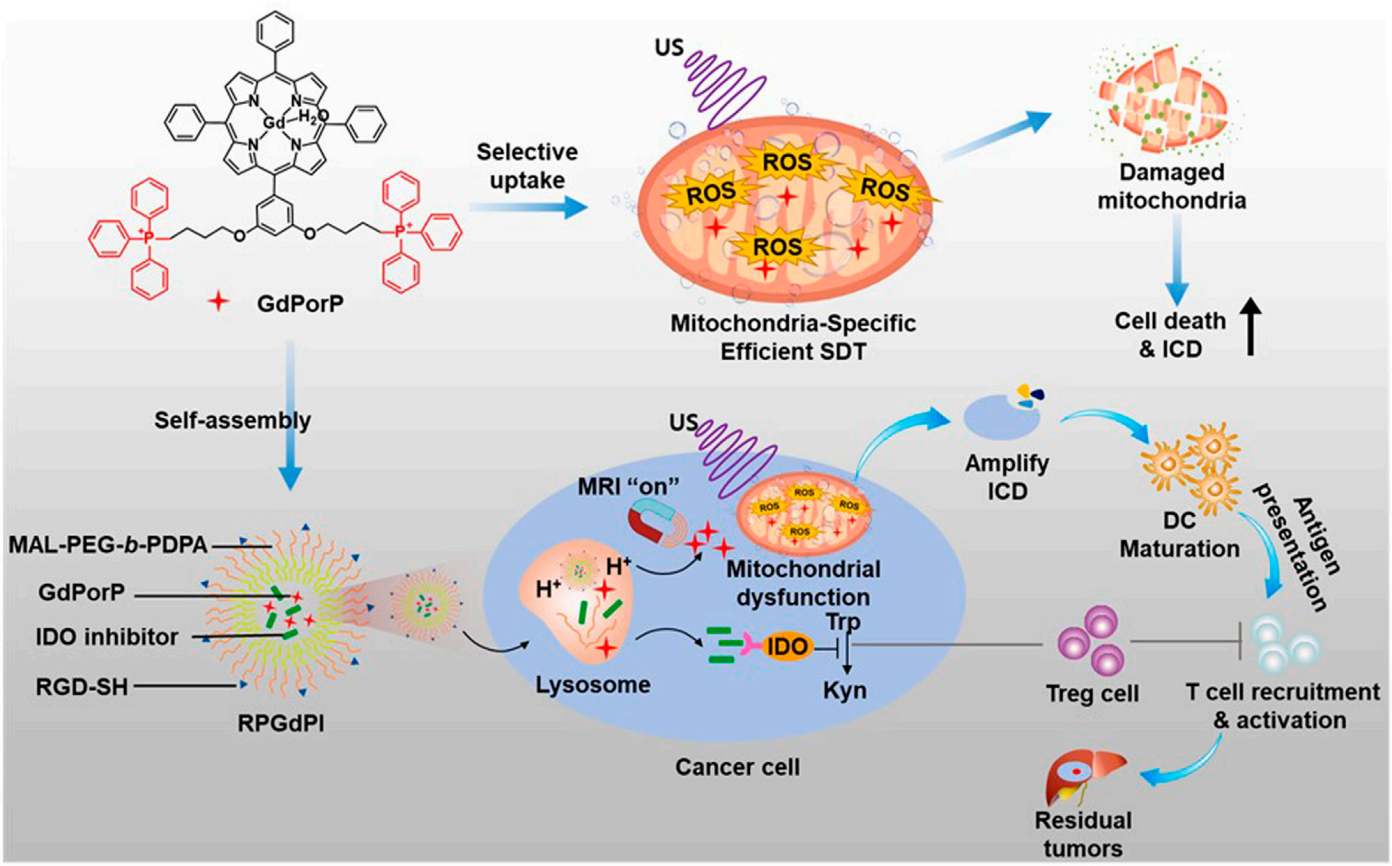
Schematic illustration of the TPP-conjugated gadolinium (III) porphyrinate (GdPorP) for high-efficiency ROS production, active mitochondrial targeting, and suitable MR contrast. Reprinted with permission from (Li et al., 2022), Copyright 2022 Elsevier.

Li et al. (2022) introduced a novel SDT agent called TPP-conjugated gadolinium (III) porphyrinate (GdPorP), which offers several advantages including high-efficiency ROS production, active mitochondrial targeting, and good MR contrast (Figure 10). To enhance tumor-targeting affinity and biocompatibility, GdPorP was coated with MAL-PEG-*b*-PDPA, forming pH-responsive nanomicelles (RPGdP). Upon delivery to H22 cancer cells, GdPorP accumulated in the cancer cell mitochondria (PC = 0.93), leading to increased cell death and amplifying ICD. Significant cell cytotoxicity was observed using RPGdP compared to the TPP-free RPGd upon the US irradiation (1 min, 1 MHz, 1.5 W/cm^2^). To further enhance the *in vivo* SDIT efficacy of this system, the authors incorporated an indolamine 2,3-dioxygenase (IDO) inhibitor within the RPGdP, resulting in a nanopharmaceutical named RPGdPI. In H22 orthotopic liver tumor mouse models, RPGdPI effectively suppressed the growth of deep-seated primary tumors and induced significant antitumor effects by reversing the immunosuppressive tumor microenvironment.

## 5 Conclusion and outlook

Mitochondria play a crucial role in energy production and are closely related to cell apoptosis. Mitochondria-targeted SDT systems offer significant advantages over traditional SDT agents by enabling precise and localized production of ROS in ROS-susceptible mitochondrial regions. These systems have demonstrated antitumor efficacy, activation of antitumor immune responses, reduced adverse effects, lower agent doses, mild US irradiation, and the ability to overcome SDT challenges such as metastasis. Mitochondria-targeted SDT agents have been extensively investigated in recent years, and some agents could bring new insights into the next SDT methods for clinical application.

US-mediated SDT agents with the mitochondria-targeting ability and efficient ROS production effect can induce increased oxidative stress in cancer cells under US irradiation. Several agents, such as HMME, IR780, Ce6, GdPorP, and PpIX derived from ALA, have demonstrated excellent efficacy in US-mediated SDT. Triphenylphosphoniums and adipocyte-derived organelles are commonly used for mitochondrial targeting in SDT. In addition, US-active agents such as IR780 and PpIX, with inherent mitochondria-targeting ability are also utilized for this purpose.

Considerable progress has been made in the development of mitochondrial SDT agents through various approaches, including US-responsive deep tumor penetration, improved cancer/mitochondria delivery with US assistance, US-related enhanced anticancer immune responses, and multimodal strategies combining SDT accompanied by other imaging-guided techniques [e.g., photoacoustic (PA) imaging, MR imaging, fluorescence (FL) imaging, etc.]. Combination therapies, such as mitochondria-targeted SDT and immunotherapy (e.g., R162), gas therapy (e.g., HSA-NO, FDC), metabolic modulation therapy (e.g., 2-DG, CPI-613), chemotherapy (e.g., PL), starvation therapy (e.g., GOx), dual SDT agent therapy (e.g., IR780/HMME, IR780/Ce6), and US-stimulated microbubble therapy (e.g., MB), are being explored to amplify the benefits of each treatment modality. However, despite the remarkable advances in the field of biomedical SDT, further development of SDT agents and proven technologies for clinical application are still needed. Clinical applications of these SDT agents are currently lacking owing to the novelty of the field.

Several challenges and areas for improvement exist in the field of SDT: 1) while mitochondria-targeted SDT has shown significant potential, there is a limited number of studies focusing on other cell organelles such as the nucleus, ER, lysosomes, and Golgi apparatus; 2) there is a need for improved methods to manufacture well-defined, organelle-targeting agents on a large scale; 3) a universal treatment approach for various types of tumors has not been established, as SDT agents generally target the general features of cancer organelles; 4) the lack of standardized methods to evaluate SDT efficacy makes it challenging to compare the properties of various types of SDT agents; 5) studies on the biocompatibility of the by-products remaining after SDT treatment with commonly used agents are still lacking.

In conclusion, there is a need for the development of more biocompatible systems using non-toxic, biodegradable agents with improved organelle targeting abilities. Further biological and pharmacokinetic studies are also necessary. As research into improved SDT agents continues, various approaches are expected to be explored for targeted SDT therapy, leading to meaningful research outcomes and potential clinical translation in the future. Continued efforts in this area hold promise for the advancement of SDT and its application in clinical settings in a foreseeable future.
